# RNA-Seq Expression Analysis of Chronic Asthmatic Mice with Bu-Shen-Yi-Qi Formula Treatment and Prediction of Regulated Gene Targets of Anti-Airway Remodeling

**DOI:** 10.1155/2021/3524571

**Published:** 2021-01-18

**Authors:** Jie Cui, Zexi Lv, Fangzhou Teng, La Yi, Weifeng Tang, WenQian Wang, Wuniqiemu Tulake, Jingjing Qin, Xueyi Zhu, Ying Wei, Jingcheng Dong

**Affiliations:** ^1^Department of Integrative Medicine, Huashan Hospital, Fudan University, Shanghai, China; ^2^Institute of Integrative Traditional Chinese and Western Medicine, Fudan University, Shanghai, China

## Abstract

Airway remodeling is one of the typical pathological characteristics of asthma, while the structural changes of the airways in asthma are complex, which impedes the development of novel asthma targeted therapy. Our previous study had shown that Bu-Shen-Yi-Qi formula (BSYQF) could ameliorate airway remodeling in chronic asthmatic mice by modulating airway inflammation and oxidative stress in the lung. In this study, we analysed the lung transcriptome of control mice and asthmatic mouse model with/without BSYQF treatment. Using RNA-sequencing (RNA-seq) analysis, we found that 264/1746 (15.1%) of transcripts showing abnormal expression in asthmatic mice were reverted back to completely or partially normal levels by BSYQF treatment. Additionally, based on previous results, we identified 21 differential expression genes (DEGs) with fold changes (FC) > (±) 2.0 related to inflammatory, oxidative stress, mitochondria, PI3K/AKT, and MAPK signal pathways which may play important roles in the mechanism of the anti-remodeling effect of BSYQF treatment. Through inputting 21 DEGs into the IPA database to construct a gene network, we inferred Adipoq, SPP1, and TNC which were located at critical nodes in the network may be key regulators of BSYQF's anti-remodeling effect. In addition, the quantitative real-time polymerase chain reaction (qRT-PCR) result for the selected four DEGs matched those of the RNA-seq analysis. Our results provide a preliminary clue to the molecular mechanism of the anti-remodeling effect of BSYQF in asthma.

## 1. Introduction

Asthma, a chronic inflammatory respiratory disease, is caused by complex factors and affects approximately 300 million people of all ages worldwide [1]. The pathogenesis of asthma is characterized by airway inflammation and airway hyperresponsiveness (AHR), and airway remodeling is the main pathophysiological feature [1, 2]. Airway remodeling is usually observed in asthma and can be driven by pathways that are partly independent of airway inflammation. It includes airway smooth muscle (ASM) mass, peribronchial collagen deposition, goblet cell, and glandular hyperplasia and angiogenesis [3, 4]. The progression of asthma is associated with the functional changes of the airways [5], whereas the complexity of airway function and structure changes in asthma has impeded the development of novel asthma targeted therapy.

Asthma-related morbidity results from immune imbalances caused by the release of inflammatory mediators including reactive oxygen species (ROS), cytokines, and growth factors [4]. ROS could induce cell damage, change the physiological function of structural cells, and play a key role in initiation as well as amplification of inflammation in asthma [6, 7]. Meanwhile, accumulating evidence has suggested that ROS is involved in airway remodeling in asthma. ROS can enhance release of transforming growth factor-*β*1 (TGF-*β*1) and vascular endothelium growth factor (VEGF), which contribute to subepithelial fibrosis and airway remodeling [6]. Mitochondria are one of the important sources of basal endogenous ROS production in the lung [8]. Furthermore, airway abnormalities, particularly secretion of various cytokines, leads to the activation of intracellular signaling pathways which are involved in airway inflammation as well as remodeling process. The PI3K/AKT pathway has been proved to play an important role in regulating cell proliferation, growth, differentiation, and metabolism [9]. As well, the mitogen-activated protein kinase (MAPK) pathway is known to regulate a variety of biological processes like cell growth and proliferation, chemotaxis, degranulation, and other processes [10]. Therefore, PI3K/AKT and MAPKs signal pathways are considered as therapeutic targets in airway remodeling of asthma.

Traditional Chinese medicines (TCMs) were derived from thousands of years of clinical use in China. TCMs containing multiple bioactive ingredients are potential novel resources for asthma treatment drugs. BSYQF is used in the clinical treatment of asthma in China and is composed of *Astragalus membranaceus* (Fisch.) Bunge, *Rehmannia glutinosa* Libosch, and *Epimedium brevicornu* Maxim. The chemical fingerprint of BSYQF contains at least 16 chemical components including icariin, acteoside, catalpol, leonuride, calycosin, and epimedin A. This indicates that multiple compounds in BSYQF may deliver an integrated anti-asthma effect through multiple targets and their associated molecular pathways.

The anti-remodeling effects of BSYQF including the inhibition of ASMC proliferation and peribronchial collagen deposition in chronic asthmatic mice have been demonstrated in our previous study [11]. We also preliminarily explored the anti-remodeling mechanism of BSYQF, which may be related to its anti-inflammatory, antioxidant, and mitochondrial structure restoration. To further investigate the molecular mechanism of BSYQF, we used lung samples obtained from previous experiments and took advantage of high-throughput whole-transcriptome analyses to explore molecular mechanisms targeted by BSYQF. BSYQF is a multicompound formula with wide molecular mechanisms. Given this complexity of multiple targets and their associated molecular pathways, increased understanding of the mechanisms of BSYQF might be best derived from the available experimental results. Hence, according to our previous result, we mainly focused on inflammatory, oxidative stress, and mitochondria in GO enrichment and PI3K/AKT and MAPKs signal pathways which are thought to play an important role in asthma airway remodeling to identify differentially expressed genes (DEGs) which may be therapeutic targets of BSYQF.

## 2. Materials and Methods

### 2.1. Animals

Lung tissue samples were obtained from our recently published study that tested the effects of BSYQF on airway remolding in murine chronic asthma [11]. Female BALB/*c* mice were sensitized and challenged with ovalbumin for 8 weeks to establish chronic asthmatic model as described previously [11]. BSYQF was prepared as described previously [12]. Briefly, *Astragalus membranaceus* (Fisch.) Bunge, *Rehmannia glutinosa* Libosch, and *Epimedium brevicornu* Maxium were decocted in the ratio of 3 : 2 : 1.5(w/w). From day 14, mice in BSYQF group were oral administrated 20g raw herbs/kg body weight, and mice in control and asthma groups were oral administrated with saline. At the end of experimental period, mice were euthanized and lung tissues used to extract RNA were harvested immediately and frozen in liquid nitrogen.

### 2.2. Tissue Sample RNA Isolation

For RNA-seq, we used 3 lung tissue samples from control mice, 3 from asthmatic mice, and 3 from BSYQF treatment mice. Total RNA was isolated using Trizol reagent as described previously [13]. The quality of RNA was checked by 1% agarose gels electrophoresis, and quantity was determined with NanoPhotometer spectrophotometer (IMPLEN, CA, USA). Optical density values were confirmed an *A*_260_: *A*_280_ ratio above 1.9. RNA integration number (RIN) was measured using the RNA Nano 6000 Assay Kit of the Bioanalyzer 2100 system (Agilent Technologies, CA, USA) to confirm RIN above 7.

### 2.3. Library Preparation and Transcriptome Sequencing

We used NEBNext^®^ Ultra^TM^ RNA Library Prep Kit for Illumina (NEB, USA) to construct sequencing libraries according to the manufacturer's recommendations. Briefly, using poly-T oligo beads, the mRNA was purified from total RNA. In NEBNext First-Strand Synthesis Reaction Buffer, fragmentation was performed using divalent cations. M-MuLV Reverse Transcriptase (RNase H) was used to synthesize the first strand cDNA, and DNA polymerase I and RNase H were used to synthesize the second-strand cDNA. Via exonuclease/polymerase activities, and remaining overhangs were transformed into blunt ends. The NEBNext adaptor with the hairpin loop structure was ligated and prepared for hybridization after the 3 ′-end of the DNA fragment was identified. Using AMPure XP system (Beckman Coulter, Beverly, USA), we purify the library fragments and the cDNA fragment with length of 250–300 bp was selected preferentially. Finally, PCR products were purified using AMPure XP system, and on the Agilent Bioanalyzer 2100 system, the library quality was evaluated. TruSeq PE Cluster Kit V3 -cBot-HS (Illumia, San Diego, CA, USA) was preformed to cluster the index coded samples according to the manufacturer's protocol on the cBot Cluster generation system. After cluster generation, sequenced library was prepared on Illumina Novaseq6000 platform to generate 150 bp paired end reads.

### 2.4. Quality Control, Mapping, and Quantification of RNA-Seq Reads

Firstly, clean reads were obtained by removing reads containing adapter or ploy-N as well as low quality reads from raw reads. Meanwhile, the Q20, Q30, and GC contents of clean data were calculated. All downstream data were analysed on the basis of high quality cleaning readings. STAR was performed to align clean reads to reference genome. In the mapping speed, STAR outperformed other aligners by a factor of >50 and improved alignment sensitivity and precision [14]. The HTSeq V0.6.0 count was mapped to the reading for each gene. The value of the FPKM (number of segments per thousand base exon; mapping per million segments) is calculated based on the length of the gene as well as the read count mapped to the gene.

### 2.5. Differential Expression Analysis

After the significant analysis and FDR analysis, only genes under |log2FC| > 1 and *p* value < 0.05 criteria were included in the analysis. DESeq2 algorithm was applied to filter the differentially expressed genes.

### 2.6. Functional Analysis

The gene ontology (GO) and Kyoto Encyclopedia of Genes and Genomes (KEGG) analysis were performed to describe the function of each DEGs. We applied the ingenuity pathway analysis (IPA, Redwood City, CA, USA) to construct gene interaction network.

### 2.7. Real-Time PCR

The primers used in this study were purchased from BioTNT (Shanghai, China). Total RNA from treated, asthma, and control lung samples was extracted by the TRIZOL method. cDNA synthesis was performed using cDNA Reverse Transcription Kit (Takara) according to the manufacturer's instructions. qRT-PCR was performed using TB Green Premix Ex Taq Kit (Takara). We used GAPDH gene as an internal reference to normalize the expression of target genes. Reactions were run on the 7500 Real-time PCR system (ABI, USA), and the data were analysed using its software. Ct (threshold cycle) was used to determine the target gene expression levels.

### 2.8. Statistical Analysis

Intergroup differences were analysed using one-way ANOVA. Data in the experiments are expressed as mean ± SD. All statistical analyses were accomplished by using PRISM version 7.0 (GraphPad). A *p* value of <0.05 was considered statistically significant.

## 3. Results

### 3.1. Gene Expression Analysis

**Figure 1:**
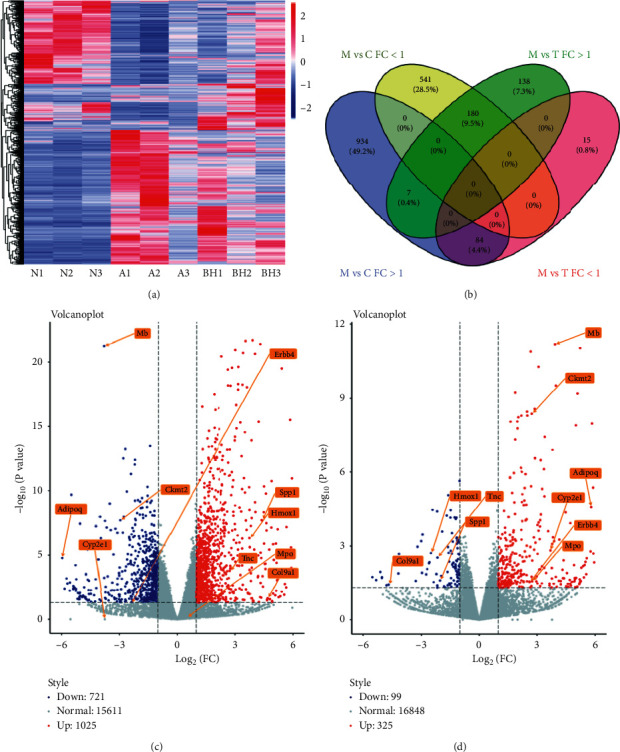
(a) The clustering heat maps of gene expressions. Red represents the upregulation expressions; blue represents the downregulation expressions. The color brightness is associated with differences in multiples. (b) The Venn diagram shows RNA-seq data for all genes that were regulated in asthma compared to control (M vs C) and by BSYQF treated compared to non-treated asthma controls (T vs M). FC: fold changes. Blue: M vs C, positive FC (FC > 1). Yellow: M vs C, negative FC (FC < 1). Green: M vs T, positive FC (FC > 1). Pink: M vs T, negative FC (FC < 1). (c). Volcano plot (M vs C). (d) Volcano plot (M vs T). Genes of interest are labeled as examples to show changes in expression levels. Horizontal dashed line represents expression value *p*=0.05. Genes outside the midline have FC > 1.

DEGseq analysis results applied on RNA-seq FPKM. The RNA expression result in lung tissue showed significant differences among control (C, control), asthma (M, model), and BSYQF treatment in the mouse model of asthma (T, treatment). As shown in Figure 1(a), clustering analysis was used to detect overexpressed genes and underexpressed genes.

### 3.2. Gene Expression Regulated by Asthma and BSYQF

Gene expression differences between the asthmatic compared to control (A vs C) and asthma compared to BSYQF treatment (A vs T) groups were shown in the Venn diagram in Figure 1(b) and Table 1. As the Venn diagram shown, changes in expression levels were observed in 1,899 transcripts in the lung. 271 (180 + 7+84 + 0) of these were regulated by both asthma and treatment. As shown in Table 1, 1,746 transcripts were regulated by asthma with log2 fold changes (FC) ranging from −8.6 to 11.7, *p* < 0.05. Of these, 1,025 were upregulated and 721 were downregulated by asthma. BSYQF treatment alters the expression of 424 transcripts with FC between −5.5 and 7.8 (*p* < 0.05). Among these, 325 were upregulated and 99 were downregulated. By BSYQF treatment, a total of 264/1746 (15.1%) transcripts including 180 upregulated and 84 downregulated transcripts, and their expression levels were reversed to normal levels.

### 3.3. Volcano Results

In Figure 1 C/D, the volcano plot shows transcriptome changes in asthmatic compared to control (A vs C) and asthma compared to BSYQF treatment (A vs T) groups in the lung tissues of mice. The scatter plot showed that the distribution of genes in significance (*y* axis, –log10 (*p* value)) vs fold changes (*x* axis, log2 (normalized fold change (FC)). As shown in Figure 1 C/*D*, genes are outside the midline of the absolute normalized FC > =(±)1, and –log10 (*p* value)> = 1.3 are colored red for overexpressed genes and blue for underexpressed genes. The underlying key genes in asthma pathogenesis and those regulated by treatment including Adipoq, HMOX1, SPP1, Cyp2e1, TNC, MB, MPO, Col9a1, Ckmt2, and Erbb4 were taken as examples to illustrate the positions and relationships with other points and midline in the figure. There were small changes in the expression levels of many genes in both directions, but only the FC > =(±)1 and *p* < 0.05 genes that met our screening criteria were meaningful in this study.

### 3.4. BSYQF Treatment Normalized Gene Expression Altered in Asthma

From this analysis (Table 1 A/B), we found a small group of genes that showed significant (above 50%) or complete reversal of changes in asthma during BSYQF therapy. These genes are listed in Table 1 C and rank-ordered by FC> =(±)5 (*p* < 0.05). These DEGs were regulated by both asthma and BSYQF and may be closely related to the mechanism of BSYQF's anti-asthma.

### 3.5. Key Gene Interaction Network Identification

**Figure 2:**
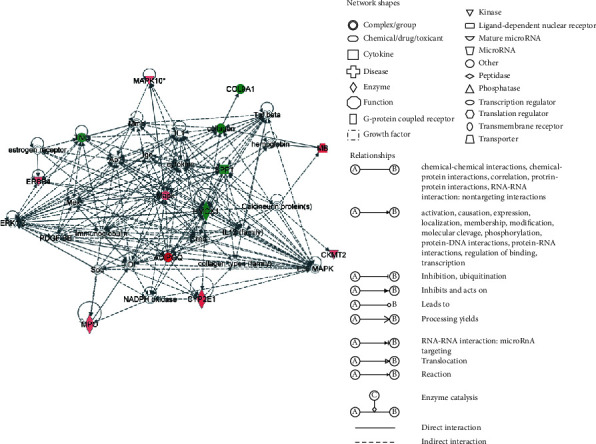
The gene networks by IPA. The intensity of green or red coloured shapes indicates the degree of down- or upregulation, respectively.

Based on the gene expression analysis and our previous results, 21 genes were selected from DEGs between asthma group and treatment group according to their relevant functions with inflammatory, oxidative stress, mitochondria, PI3K/AKT, and MAPKs signal pathways which are thought to be related to the action mechanism of BSYQF (Table 2). Then, we applied IPA software to identify gene networks about 21 DEGs (Figure 2). 11 of 21 input genes could be located in this network including Adipoq, HMOX1, SPP1, Cyp2e1, TNC, MB, MPO, Col9a1, Ckmt2, Erbb4, and MAPK10. In the network, Adipoq, HMOX1, SPP1, and TNC appeared to be key components which directly or indirectly interact with multiple genes and participate in multiple biological functions.

### 3.6. Verification of Selected DEGs

**Figure 3:**
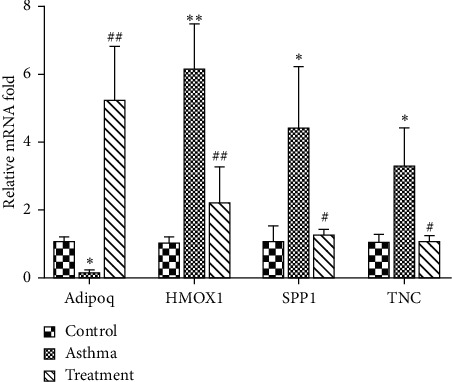
qRT-PCR validation of Adipoq, HMOX1, SPP1, and TNC results. ^*∗*^*p* < 0.05, ^*∗∗*^*p* < 0.01 vs control; ^#^*p* < 0.05, ^##^*p* < 0.01 vs asthma.

According to IPA analysis result, Adipoq, HMOX1, SPP1, and TNC were selected for further validation. We performed qRT-PCR analysis on the four genes to confirm the results of DEGs in the lung. As shown in Figure 3, a significant reduction of adiponectin (Adipoq) gene expressions in asthma group compared with control group were observed. In addition, the expressions of heme oxygenase 1 (HMOX1), tenascin C (TNC), and secreted phosphoprotein 1 (SPP1) in asthma group compared with control group genes were significantly elevated, while BSYQF treatment completely or partially reversed asthma-induced expression changes of Adipoq, HMOX1, SPP1, and TNC. The results showed that the expression of the four genes from qRT-PCR was consistent with the RNA-seq analysis pattern.

## 4. Discussion

In this research, we identified a set of transcriptome changes in the lung of chronic asthmatic mice with and without BSYQF treatment. Lung tissues were taken from our recently published study in which we have demonstrated that BSYQF treatment could ameliorate airway remodeling in asthmatic mice by reducing airway inflammation and oxidative stress in the lung [11]. The results from this study provided us insight into the molecular mechanism of BSYQF's anti-airway remodeling in chronic asthmatic mice.

We identified genes whose expression in asthmatic mice were impacted by BSYQF treatment. 264/1746 (15.1%) of genes showing abnormal expression in asthma group were reverted back to completely or partially to normal levels by BSYQF treatment. These included Adipoq, also known as Adiponectin, which in this study was found to be one of the most prominently downregulated genes in asthma and completely reversed back by BSYQF treatment. It has been shown that Adiponectin is an anti-inflammatory adipokine [15]. Since BSYQF has been shown to effectively regulate many pathological processes in asthma, we proposed that this core set of genes may be related to the pathology of asthma and represent key BSYQF targets.

Based on previous result, we identified a set of genes related to inflammatory, oxidative stress, mitochondria, PI3K/AKT, and MAPKs signal pathways which may play important roles in the mechanism of BSYQF's anti-remodeling. We screened 21 DEGs with FC > (±) 2.0 that were differentially expressed between asthma and treatment. Additionally, 21 DEGs were input into IPA database to obtain gene networks related to regulatory functions. Gene network analyses by IPA are based on current known pathways. We used IPA because we aimed to elucidate the interaction of the gene expression found in specific functions and pathways which are thought to be related to BSYQF's anti-remodeling. Furthermore, we identified the possible key targets of BSYQF through IPA. In the gene network analyses by IPA, we found that Adipoq, HMOX1, SPP1, and TNC are genes at critical nodes. We also verified the four DEGs selected through qRT-PCR and confirmed that their variation trend were consistent with the RNA-seq analysis.

Adipoq, also known as diponectin, is an important adipokine with anti-inflammatory and antioxidative effects [16]. Adiponectin plays a physiological role by activating receptors such as AdipoR1, AdipoR2, and T-cadherin which are expressed on airway epithelial and endothelial pulmonary cells playing functional roles on lung physiology [17, 18]. AdipoRs mediate pleiotropic adiponectin actions through signaling mechanisms including AMPK, AKT, ERK1/2, and P38 [19]. Previous study reported that adiponectin was reduced in the asthmatic murine model [20]. Additionally, adiponectin attenuated the airway inflammation and AHR in asthmatic mice [20]. Another study has also shown that adiponectin can reduce inflammation and suppress oxidative stress in vivo [21]. Adiponectin not only downregulates proinflammatory cytokines including TNF-*α*, IL-6, and NF-*κ*B but also upregulates anti-inflammatory cytokines such as IL-10 [22]. Benjamin *D* et al. found that adiponectin deficiency increased allergic airway inflammation and pulmonary vascular remodeling [23]. In our RNA-seq result, Adipoq was significantly downregulated more than five-fold in asthma group and reversed completely after BSYQF treatment which drew our attention. The qRT-PCR results further confirmed the result of RNA-seq. Furthermore, in the gene network analysed by IPA, Adipoq interacted with multiple genes and participated in multiple cellular functions. Hence, the mechanism related to Adipoq expression might provide a new understanding for illustrating the therapeutic target of BSYQF's anti-remodeling.

HOMX1, known as HO-1, is a rate-limiting enzyme that catalyzes the degradation of heme into biliverdin, ferrous ion, and carbon monoxide [24]. Studies have shown that HO-1 has anti-inflammatory, excessive cell proliferation and interstitial fibrosis effects [25]. Previous study has shown that HO-1 could ameliorate airway inflammation by downregulating the tumor necrosis factor receptor (TNFR)1 dependent oxidative stress [26]. In asthma, increased HO-1 expression and activity are considered protective. The expression level of HO-1 was associated with its anti-asthma effect. Our results turned out that HO-1 gene expression was increased in asthmatic mice which was consistent with that in the previous study [27]. However, there are conflicting results about the role of BSYQF in regulating the HO-1 expression. We observed BSYQF treatment reduced HO-1 gene expression by using RNA-seq analysis and qRT-PCR. This implies that it is possible that the effect of BSYQF on HO-1 expression differs between gene and protein levels and further studies are needed.

SPP1 (secreted phosphoprotein 1, also known as osteopontin) is a phosphorylated glycoprotein secreted by activated macrophages, leueocytes, and activated T lymphocytes [28]. It is recognized as a key cytokine involved in Th1 cytokine expression and immune cell recruitment at sites of inflammation [28, 29]. It is also a mediator of tissue repair and remodeling [30, 31]. According to RNA-seq and qRT-PCR results, we found the SPP1 expression increased in asthma and decreased after BSYQF treatment which suggested that SPP1 might play important effect on the mechanism of BSYQF's anti-remodeling.

TNC is considered an important gene in asthma pathogenesis [32] and known as an extracellular matrix (ECM) protein whose expression is increased in asthma [32]. TNC plays important roles in cell communication and signal transduction and regulates cell adhesion, migration, proliferation, and differentiation [32, 33]. Study in TNC knockout (KO) mice showed that lung disease phenotype was largely attenuated in the absence of TNC in OVA-induced asthma [34]. Our RNA-seq and qRT-PCR results confirmed that TNC up-regulation in asthma. Additionally, we found that TNC mRNA expression decreased after BSYQF treatment. These results provided that BSYQF may have a beneficial effect on asthma by reducing the TNC expression.

## 5. Conclusion

In this study, we used RNA-seq analysis to obtain the differentially expressed genes. We found expressions of a group of coding genes were altered in asthmatic mice. Treatment with BSYQF reversed the expressions of a specific subset of these genes in asthmatic mice. As BSYQF was previously shown to ameliorate airway remodeling by reducing airway inflammation and oxidative stress in asthmatic mice, we enriched a group of genes related to inflammatory, oxidative stress, mitochondria, PI3K/AKT, and MAPKs signal pathways and used IPA to construct gene network. Based on gene network, we inferred that Adipoq, SPP1, and TNC may be key regulators of BSYQF's anti-remodeling effect. While RNA-seq analysis could not show protein levels and the precise role of these genes needs further verification, they provided initial clues to explore the mechanisms of BSYQF's anti-remodeling in asthma.

## Figures and Tables

**Table 1:** Differently expressed genes showing the highest fold changes (in either direction) in lung samples of the mice in the control (C), asthmatic model (*M*), and BSYQF treatment (*T*) groups.

A. M vs C		B. T vs M		C		
Gene	Fold change	Gene	Fold change	Gene	M vs C	T vs M
*Increased*		*Increased*		Timp1	6.22	−3.01
Chil4	11.74	A530016L24Rik	7.79	Reg1	6.20	−3.30
Sprr2a3	11.18	Tmem179	7.17	Dmp1	5.65	−4.15
Clca1	9.86	Serpinb11	7.01	Glod5	5.55	−5.52
Mcpt1	8.71	Ebf2	6.85	Rnf17	5.35	−5.33
Rnase2a	8.43	Htr3a	6.59	Dspp	5.14	−5.11
Capn9	7.48	Lep	6.52	Lep	−8.64	6.52
Saa3	7.42	Glycam1	6.43	A530016L24Rik	−8.36	7.79
Saa1	7.23	Syt4	6.39	Tmem179	−7.57	7.17
Saa4	7.18	Lrtm1	5.95	Lctl	−7.36	5.20
Retnlb	7.09	Nalcn	5.91	Ebf2	−6.77	6.85
A	6.93	Slc5a8	5.89	Nalcn	−6.42	5.91
Cd209e	6.93	Dsc3	5.84	Adipoq	−5.97	5.80
Mmp12	6.84	Adipoq	5.80	Htr3a	−5.58	6.59
Timd2	6.78	Lsmem1	5.76	Myh1	−5.57	4.94
Retnla	6.64	Ugt2b34	5.57	Car3	−5.50	5.10
Cbln1	6.47	Cartpt	5.54	Gm10382	−5.40	4.72
Mrgprg	6.32	Dnmt3c	5.51	Lrtm1	−5.39	5.95
Prss32	6.27	Nefm	5.46	Slc5a8	−5.29	5.89
Timp1	6.22	Slc38a3	5.42	Hist1h4k	−5.27	5.29
Reg1	6.20	Pnoc	5.39	Kcnq3	−5.26	5.22
Fgl1	6.18	Hist1h4k	5.29	Pnoc	−5.18	5.39
Btbd16	6.12	Krt6a	5.26	Dnmt3c	−5.16	5.51
Nuggc	6.11	Serpina1e	5.24	Retn	−5.09	4.96
*Decreased*		Acp7	5.24	Cfd	−5.06	5.01
Lep	−8.64	Krt5	5.23	Lsmem1	−5.03	5.76
A530016L24Rik	−8.36	Kcnq3	5.22	—	—	
Tmem179	−7.57	BC117090	5.21			
Lctl	−7.36	Lctl	5.20			
Ebf2	−6.77	BC016579	5.19			
Gys2	−6.69	Car3	5.10			
Ebf3	−6.58	Gm5416	5.09			
Nalcn	−6.42	Dync1i1	5.03			
—	—	Cfd	5.01			
		*Decreased*				
		Glod5	−5.52			
		Rnf17	−5.33			
		Dspp	−5.11			
		Gm49320	−5.03			
		Azgp1	−5.03			

A, all genes with increased or decreased expression levels by FC > (±)6 (*p* < 0.05) in the asthma group (M) relative to the control group (C); B, all genes upregulated or downregulated by the BSYQF treatment group (*T*) with FC > =(±)5 (*p* < 0.05) relative to the asthma group (M). C, all genes with FC > (±)5 (*p* < 0.05) that were reverted in their expression towards normal levels by BSYQF treatment. Genes are ranked orderly by FC (positive or negative values).

**Table 2:** Differently expressed gene with FC > (±) 2.0 in asthma group (*M*) relative to treatment group (*T*) related to inflammatory, oxidative stress, mitochondria, PI3K/AKT, and MAPK signal pathways.

Gene name	Description	log2 fold change (A vs T)
Adipoq	Adiponectin C1Q and collagen domain containing	5.80
Nefm	Neurofilament medium polypeptide	5.46
Car3	Carbonic anhydrase 3	5.10
Mb	Myoglobin	3.94
Krt15	Keratin 15	3.99
Cyp2e1	Cytochrome P450 family 2 subfamily e polypeptide 1	3.71
Gdap1	Ganglioside induced differentiation associated protein 1	3.66
Cyp4a12b	Cytochrome P450 family 4 subfamily a polypeptide 12B	3.10
Cacna2d2	Calcium channel voltage dependent alpha 2 delta subunit 2	2.77
Mpo	Myeloperoxidase	2.77
Ckmt2	Creatine kinase mitochondrial 2	2.75
Erbb4	Erb-B2 receptor tyrosine kinase 4	2.63
Mapk10	Mitogen-activated protein kinase 10	2.47
Pln	Phospholamban	2.42
Obscn	Obscurin cytoskeletal calmodulin and titin interacting RhoGEF	2.24
Cox8b	Cytochrome c oxidase subunit VIIIb	2.23
Col9a1	Collagen type IX alpha 1	−4.69
Mt-Nd4l	Mitochondrially encoded NADH dehydrogenase 4L	−2.94
Hmox1	Heme oxygenase 1	−2.48
Tnc	Tenascin C	−2.16
Spp1	Secreted phosphoprotein 1	−2.02

## Data Availability

The data used to support the findings of this study are available from the corresponding author upon request.
